# Identifying hub genes in response to ustekinumab and the impact of ustekinumab treatment on fibrosis in Crohn’s disease

**DOI:** 10.3389/fimmu.2024.1401733

**Published:** 2024-05-22

**Authors:** Ying Xu, Shu Wang, Ziping Ye, Hongjie Zhang

**Affiliations:** Department of Gastroenterology, The First Affiliated Hospital with Nanjing Medical University, Nanjing, Jiangsu, China

**Keywords:** Crohn’s disease, ustekinumab, treatment response, intestinal fibrosis, transcriptomics

## Abstract

**Introduction:**

Crohn’s disease (CD) is a chronic inflammatory disease. Approximately 50% of patients with CD progressed from inflammation to fibrosis. Currently, there are no effective drugs for treating intestinal fibrosis. Biologic therapies for CD such as ustekinumab have benefited patients; however, up to 30% of patients with CD have no response to initial treatment, and the effect of ustekinumab on intestinal fibrosis is still uncertain. Therefore, it is of great significance to explore the predictive factors of ustekinumab treatment response and the effect of ustekinumab on intestinal fibrosis.

**Materials and methods:**

Public datasets—GSE207465 (blood samples) and GSE112366 and GSE207022 (intestinal samples)—were downloaded and analyzed individually (unmerged) based on the treatment response. Differentially expressed genes (DEGs) were identified by the “limma” R package and changes in immune cell infiltration were determined by the “CIBERSORT” R package in both blood and intestinal samples at week 0 (before treatment). To find predictive factors of ustekinumab treatment response, the weighted gene co-expression network analysis (WGCNA) R package was used to identify hub genes in GSE112366. Hub genes were then verified in GSE207022, and a prediction model was built by random forest algorithm. Furthermore, fibrosis-related gene changes were analyzed in ileal samples before and after treatment with ustekinumab.

**Results:**

(1) Our analysis found that *MUC1*, *DUOX2*, *LCN2*, and *PDZK1IP1* were hub genes in GSE112366. GSE207022 revealed that *MUC1* (AUC:0.761), *LCN2* (AUC:0.79), and *PDZK1IP1* (AUC:0.731) were also lower in the response group. Moreover, the random forest model was shown to have strong predictive capabilities in identifying responders (AUC = 0.875). To explore the relationship between intestinal tissue and blood, we found that *ITGA4* had lower expression in the intestinal and blood samples of responders. The expression of *IL18R1* is also lower in responders’ intestines. *IL18*, the ligand of *IL18R1*, was also found to have lower expression in the blood samples from responders vs. non-responders. (2) GSE112366 revealed a significant decrease in fibrosis-related module genes (*COL4A1*, *TUBB6*, *IFITM2*, *SERPING1*, *DRAM1*, *NAMPT*, *MMP1*, *ZEB2*, *ICAM1*, *PFKFB3*, and *ACTA2*) and fibrosis-related pathways (ECM–receptor interaction and PI3K-AKT pathways) after ustekinumab treatment.

**Conclusion:**

*MUC1*, *LCN2*, and *PDZK1IP1* were identified as hub genes in intestinal samples, with lower expression indicating a positive prediction of ustekinumab treatment response. Moreover, *ITGA4* and *IL18/IL18R1* may be involved in the treatment response in blood and intestinal samples. Finally, ustekinumab treatment was shown to significantly alter fibrotic genes and pathways.

## 1 Introduction

Crohn’s disease (CD) is a chronic inflammatory gastrointestinal disease with recurrence, progression, and disability (1). CD was first described in 1932, and the incidence is rising now, but the pathogenesis is still poorly understood (2). Existing studies have shown that CD is related to genetic factors, triggered by environmental factors, and associated with immune disorders (i.e., imbalance of effector and regulatory T cells and cytokines, and migration and retention of leukocytes) (3). Patients with CD were divided into three groups by disease behavior (non-stenosis and non-penetrating, stenosis, and penetrating) according to the Montreal classification. Approximately 50% of patients with CD experienced progression from inflammation to fibrosis, which leads to intestinal stenosis and even bowel obstruction (4). Intestinal strictures in patients with CD often require surgery and seriously affect patients’ quality of life (4). The medications used to treat CD include mesalazine, methotrexate, thiopurines, and biologic therapies, such as antibodies to tumor necrosis factor alpha (TNF-α), interleukin (IL)-12/23, and integrin α4β7 (1, 5). Biologic therapies have benefited patients with CD, but there is still a therapeutic ceiling and some patients do not respond to some biologic therapies.

Ustekinumab, human monoclonal IL-12/23 p40 antibody, is a new biological agent and is approved for the treatment of patients with moderate-to-severe CD (6). Inflammatory changes in CD are related to an imbalance between Th1, Th17, and Treg cells. Moreover, IL-12 is responsible for the differentiation of naive T helper cells into Th1 cells and IL-23 is important for the proliferation of Th17 cells (7). Many studies have shown the efficacy of ustekinumab in patients with CD (8–10). Treatment response includes clinical response, biological response, and endoscopic healing. Clinical remission is a short-term goal, while endoscopic remission and mucosal healing are long-term goals (11). However, up to 30% of patients do not respond to initial treatment (12). Therefore, finding factors that can predict which patients will respond to ustekinumab and which will not is essential in expediting effective patient treatment.

Currently, no globally accepted predictive factors have been identified to determine if a patient will be a responder or non-responder with ustekinumab treatment. We used datasets from the public Gene Expression Omnibus (GEO) database to analyze gene alterations between the responders and non-responders with ustekinumab treatment. In addition, intestinal fibrosis seriously affects the quality of life of patients. Currently, there are no effective drugs for treating intestinal fibrosis. One study has shown that the intestinal wall thickness in patients with CD significantly improved after treatment with infliximab and ustekinumab; however, the shear wave velocity index only in the ustekinumab group significantly decreased after treatment (13). Shear wave velocity index is a measurement that can reflect the hardness and the fibrosis degree of a tissue. It is measured by intestinal ultrasound to evaluate the scissoring speed of a shear wave induced by an acoustic radiation force impulse (13). Does ustekinumab have an impact on fibrosis? We wanted to elucidate the impact of ustekinumab on fibrosis by analyzing publicly available datasets before and after treatment with ustekinumab.

## 2 Materials and methods

### 2.1 Data downloaded from the GEO database

**Figure 1:**
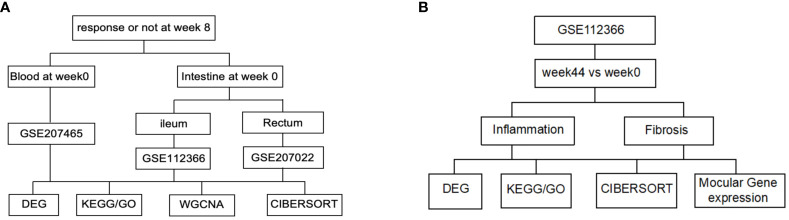
Flow diagram of the data analysis. **(A)** Analysis of the baseline index. **(B)** Analysis of the index before and after ustekinumab treatment.

Using the keywords “Ustekinumab” and “Crohn’s disease” to search on the GEO database, three datasets were downloaded based on the presence of clinical data on treatment response (**Table 1**). GSE207465 (Swati V et al, published,2022) reflects gene expression in peripheral blood, while GSE112366 (VanDussen KL et al, published,2019) and GSE207022 (Pavlidis P et al, published, 2022) reflect gene expression in the intestine (**Figure 1A**). In these datasets, RNA was collected before treatment, week 0, and then RNA samples were collected 8 weeks later. Clinical data were used to identify responders from non-responders posttreatment. The responders in GSE112366 and GSE207465 were defined as patients who experienced a decrease of 100 points from their baseline value or a value <150 by the Crohn’s disease activity index (CDAI) (clinical response). The treatment response in GSE207022 was defined as mucosal healing. We also analyzed the gene expression alterations before and after treatment with ustekinumab by GSE112366 (**Figure 1B**).

**Table 1:** Accession information for GEO datasets.

Accession	GPL	Treatment	Disease
GSE207465	GPL32416	Ustekinumab	Crohn’s disease
GSE112366	GPL13158	Ustekinumab	Crohn’s disease
GSE207022	GPL13158	Ustekinumab	Crohn’s disease

### 2.2 Identification of differentially expressed genes

Bulk RNA-seq analysis was performed using R to determine differentially expressed genes (DEGs) between ustekinumab responders and non-responders. The “limma” R package (14) was used to detect DEGs using *p* < 0.05, and the mean Log2FC plus 2 standard deviations as the cutoff values. The results were visualized using the “ggplot2” and “pheatmap” R packages.

### 2.3 Enrichment analysis of KEGG and GO

The R packages “clusterProfiler” (15) and “org.Hs.eg.db” were used to perform functional analyses. Gene Ontology (GO) and Kyoto Encyclopedia of Genes and Genomes (KEGG) enrichment analyses were performed on DEGs using ***q***-value < 0.05 as a threshold. GO terms focus on the cell function. It includes three factors: biological process (BP), cellular component (CC), and molecular function (MF) (16). KEGG was used to analyze the signaling pathways of DEGs. The data were shown in a bar chart or bubble chart using “ggplot2”.

### 2.4 Weighted gene co-expression network analysis and hub genes

We performed the weighted gene co-expression network analysis (WGCNA) using the WGCNA R package (17, 18). Then, we used the function pickSoft Threshold to select an appropriate soft power β. To identify the clinical characteristics, we created a topological overlap matrix (TOM) containing module assignments that were labeled by color and module eigengenes (ME). In addition, Pearson correlation coefficients were calculated to evaluate the correlation between ME and clinical characteristics (19). We then identified protein–protein interaction (PPI) networks using the STRING database (http://string-db.org). The image of the STRING database was imported into the Cytoscape and the cytoHubba plugin was used to predict hub genes.

### 2.5 Immune cell infiltration estimation (CIBERSORT)

The CIBERSORT algorithm was used to evaluate the percentage of 22 immune cell types (20) and results were presented using the “ggboxplot” R package. Wilcoxon tests were used to compare cell proportions between two groups. The correction between genes and immune cells was evaluated with the “psych” R package, and the results were visualized by the “ggcorrplot” R package.

### 2.6 Random forest model

Datasets were split into a training dataset (TrS) with 70% of the data and an independent test dataset (InT) with 30% of the data by the “caret” R package (21). Then, the RF algorithm was used to learn the data patterns in TrS using the “randomForest” R package. The RF model was constructed with a gradually decreasing number of important features by continuous exclusion of features with low importance. After the model was built, InT was used to test the performance of the model. The sensitivity and specificity of these models were evaluated using the ratios of true positive (TP), false positive (FP), true negative (TN), and false negative (FN) of the model. In addition, an ROC curve was plotted using the R package pROC (v1.18) (22), and the area under the curve (AUC) was also calculated to evaluate the model performance.

### 2.7 Establishment of transcription factor and miRNA regulatory network of hub genes

This study used the JASPAR database (23) to predict the transcription factor regulation network for hub genes and used the TarBase database (24) to predict the miRNA regulation network for hub genes through NetworkAnalyst (https://www.networkanalyst.ca/) (25). The results were imported into Cytoscape software for visualization.

### 2.8 Statistical analysis

R software was used for all statistical data analysis. Student’s *t*-tests or Wilcoxon tests were used to compare two groups. Correlation analysis was assessed using Pearson correlation. Predictive biomarkers were evaluated using ROC curve analysis. *p*-values < 0.05 were considered statistically significant.

## 3 Results

### 3.1 DEGs in GSE112366 between response and non-response group

**Figure 2:**
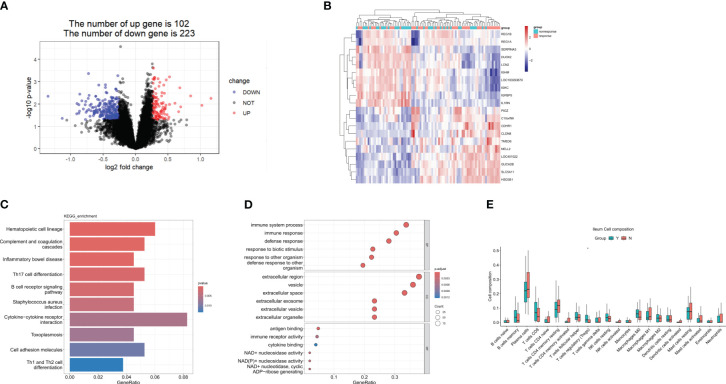
DEGs in the response group compared to the nonresponse group in the ileum at week 0. **(A)** A volcanic map of the DEGs in GSE112366. **(B)** A heatmap of the top 10 upregulated and the top 10 downregulated DEGs. **(C)** The KEGG analysis. **(D)** The top 10 functional enrichment in BP, CC, and MF analysis. **(E)** Differences in immune cell infiltration between the response group and the non-response group. Y: response, N: non-response. DEGs, differentially expressed genes; KEGG, Kyoto Encyclopedia of Genes and Genomes. Wilcoxon tests were used for statistics. *p < 0.05.

GSE112366 was downloaded to compare the baseline gene expression in ileal tissues between ustekinumab responders and non-responders. Thirty-eight samples from non-responders and 48 samples from responders were included, while the samples from patients who received placebo and without CD were excluded. A total of 345 DEGs were identified, of which 102 genes were upregulated and 223 were downregulated (**Figure 2A**). The top 10 upregulated genes and 10 downregulated genes are shown in a heatmap in **Figure 2B**. KEGG analysis revealed that DEGs were enriched in Th17 cell differentiation, B-cell receptor signaling, cytokine–cytokine receptor interaction, and Th1 and Th2 cell differentiation pathways (**Figures 2C, D**). Comparison of immune cell infiltration between two groups indicated that the percentage of Treg cells was higher in responders compared to non-responders (**Figure 2E**).

### 3.2 Identification of the hub genes in ileal samples between the response and non-response group

**Figure 3:**
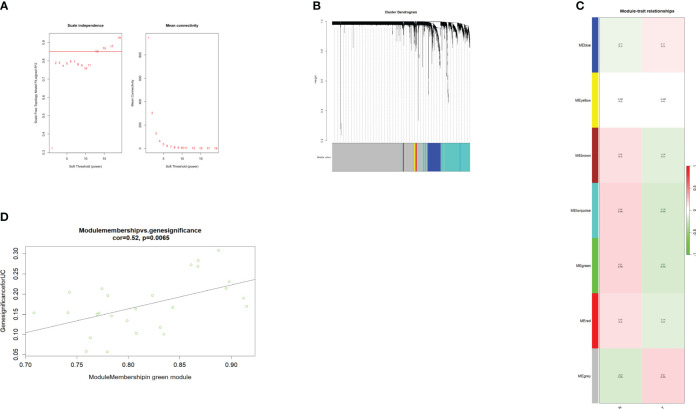
WGCNA of the GSE112366 dataset. **(A)** The soft threshold power of WGCNA. The power was 13. **(B)** The genes with strong correlation were clustered into the same module, and different modules were represented by different colors. **(C)** The correlation between the modules and the treatment response. **(D)** The green module was significantly correlated with the treatment response (COR = 0.52, *p* = 0.0065). WGCNA, weighted gene co-expression network analysis.

**Figure 4:**
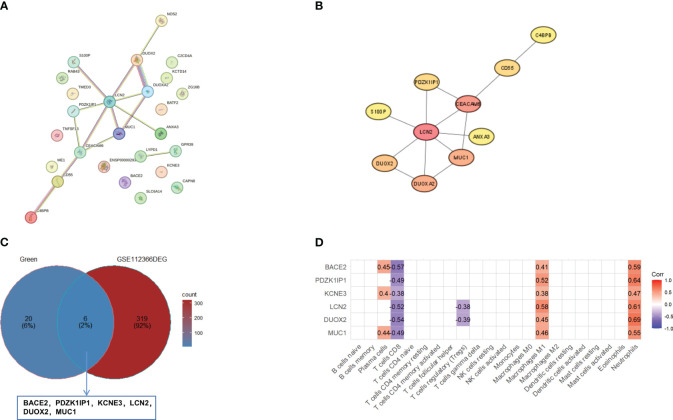
Identification of hub genes. **(A)** The PPI network of genes in green module in the STRING database. **(B)** The top 10 genes were evaluated by the cytoHubba plugin. **(C)** The Venn diagram of overlapping between the genes in the green module and DEGs in GSE112366. The overlapping genes are *BACE2*, *PDZK1IP1*, *KCNE3*, *LCN2*, *DUOX2*, and *MUC1.* **(D)** The correlation between overlapping genes and 22 immune cells. Pearson correlations were used for statistics. DEGs, differentially expressed genes.

To find the key genes, we used WGCNA to build a weight co-expression network (**Figures 3A, B**) and the green module was significantly associated with clinical feature (*r* = 0.52, *p* = 0.0065) (**Figures 3C, D**). Furthermore, genes in the green module were used to construct a PPI network in the STRING database (**Figure 4A**). In addition, the PPI data were imported into the Cytoscape software for hub gene identification by the cytoHubba plugin (**Figure 4B**). The top 10 genes were *LCN2*, *CEACAM6*, *MUC1*, *DUOXA2*, *DUOX2*, *CD55*, *PDZK1IP1*, *S100P*, *ANXA3*, and *C4BPB* (**Table 2**). After taking the intersection of DEGs of GSE112366 and the green module from WGCNA (**Figure 4C**), we concluded that *MUC1*, *DUOX2*, *LCN2*, and *PDZK1IP1* were hub genes that have lower expression in responders compared to non-responders. The expression of these genes was mainly associated with Treg cells, M1 macrophages, and neutrophils (**Figure 4D**).

**Table 2:** The top 10 hub genes in the green module genes of ileum evaluated by cytoHubba.

Rank	Name	Score
1	LCN2	10
2	CEACAM6	5
3	MUC1	4
3	DUOXA2	4
5	DUOX2	3
6	CD55	2
6	PDZK1IP1	2
8	S100P	1
8	ANXA3	1
8	C4BPB	1

### 3.3 Prediction efficiency verification of hub genes using GSE207022

**Figure 5:**
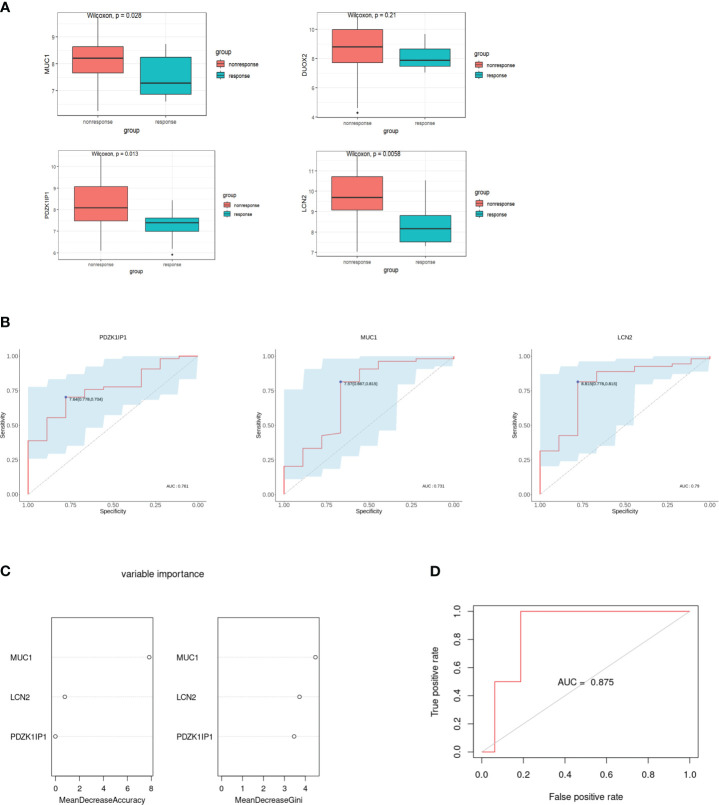
Verification of hub genes and establishment of the random forest model. **(A)** Comparison of the expression of hub genes in the GSE207022 dataset between the response and the non-response group. **(B)** The area under ROC curve indicates the effectiveness of the hub genes in prediction of treatment response to ustekinumab in the GSE207022 dataset. **(C)** Relative importance of all features in the current study based on mean decrease in accuracy (left) and mean decrease in Gini index (right) in the GSE207022 dataset. **(D)** The ROC plot for the random forest model in the GSE207022 dataset, AUROC is 0.875.

GSE207022 was downloaded to verify hub genes. We used the “limma” package to determine the DEGs of GSE207022 in the rectal mucosa of patients with CD at baseline based on response to ustekinumab treatment at week 8. A total of 550 DEGs were identified, among which 277 were upregulated and 273 were downregulated. Moreover, *MUC1*, *LCN2*, and *PDZK1IP1* were also lower in the rectum of responders versus non-responders (**Figure 5A**). The AUC–ROC analysis showed that the area under the receiver operating characteristic (AUROC) of *MUC1* was 0.731 (95% CI: 0.518–0.945) with a specificity of 66.7% and a sensitivity of 81.5%. The AUROC of *LCN2* was 0.79 (95% CI: 0.619–0.962) with a specificity of 77.8% and a sensitivity of 81.5%. For *PDZK1IP1*, the AUROC was 0.761 (95% CI: 0.612–0.911) with a specificity of 77.8% and a sensitivity of 70.4% (**Figure 5B**). The AUC–ROC curve analysis indicated that *MUC1*, *LCN2*, and *PDZK1IP1* had a good performance in predicting response to ustekinumab.

For better prediction, we built an RF prediction model based on the expression of *MUC1*, *LCN2*, and *PDZK1IP1*; GSE207022 was randomly split into a training dataset (TrS) with 70% data and an independent test dataset (InT) with 30% data. Moreover, both mean decrease in accuracy and mean decrease in Gini index showed that *MUC1* played the most important role in this model (**Figure 5C**). The RF model exhibited high efficiency to distinguish the ustekinumab responders from the non-responders (AUROC = 0.875) (**Figure 5D**).

**Figure 6:**
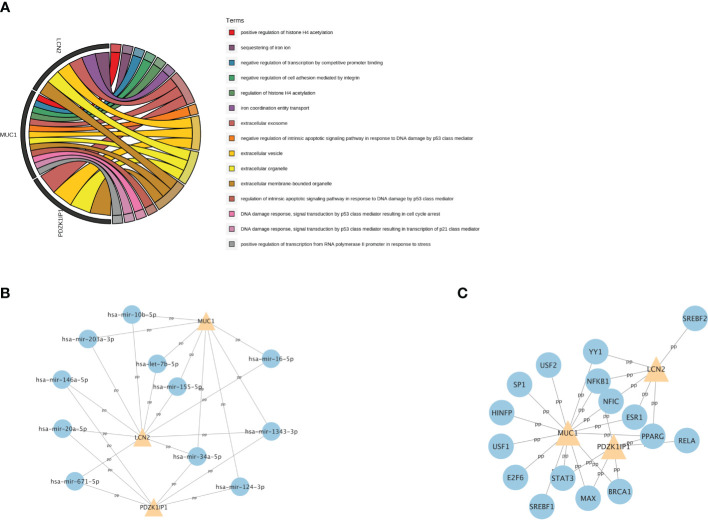
The GO analysis and the related regulatory network of hub genes. **(A)** The top 15 GO terms with the greatest significance of three hub genes. GO: Gene Ontology. **(B)** The network of transcription factors and three hub genes. **(C)** The network of miRNAs and three hub genes.

Then, we explored the function of these three hub genes. GO analysis showed that *MUC1* is mainly enriched in DNA damage and repair, *LCN2* is mainly enriched in iron metabolism, and they are all enriched in extracellular membrane-bounded organelle (**Figure 6A**).

To further understand the relevant regulatory networks of the hub genes, we used the TarBase and the JASPAR databases to predict the relevant regulatory networks of miRNAs (**Figure 6B**) and transcription factors (TFs) (**Figure 6C**). Eleven miRNAs and 16 TFs were predicted. Among them, NFKB1 and PPARG were co-transcription factors that regulate these three hub genes. In addition, miR-34a-5p and miR-1343–3p were co-miRNAs.

### 3.4 DEGs in intestinal and blood samples between the response and non-response group

**Figure 7:**
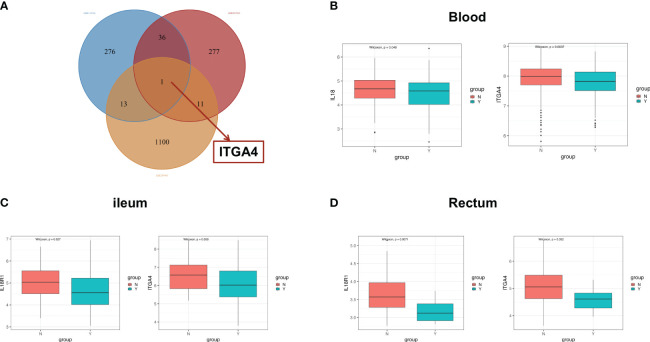
The co-expression genes in ileal and blood samples. **(A)** The Venn diagram of overlapping DEG genes among the GSE112366, GSE207022, and GSE207465 datasets. The difference of the expression of *ITGA4* and *IL18R1* between the response group and the nonresponse group in the blood **(B)**, ileum **(C)**, and rectum **(D)**. DEGs, differentially expressed genes; Y: response, N: non-response.

**Figure 8:**
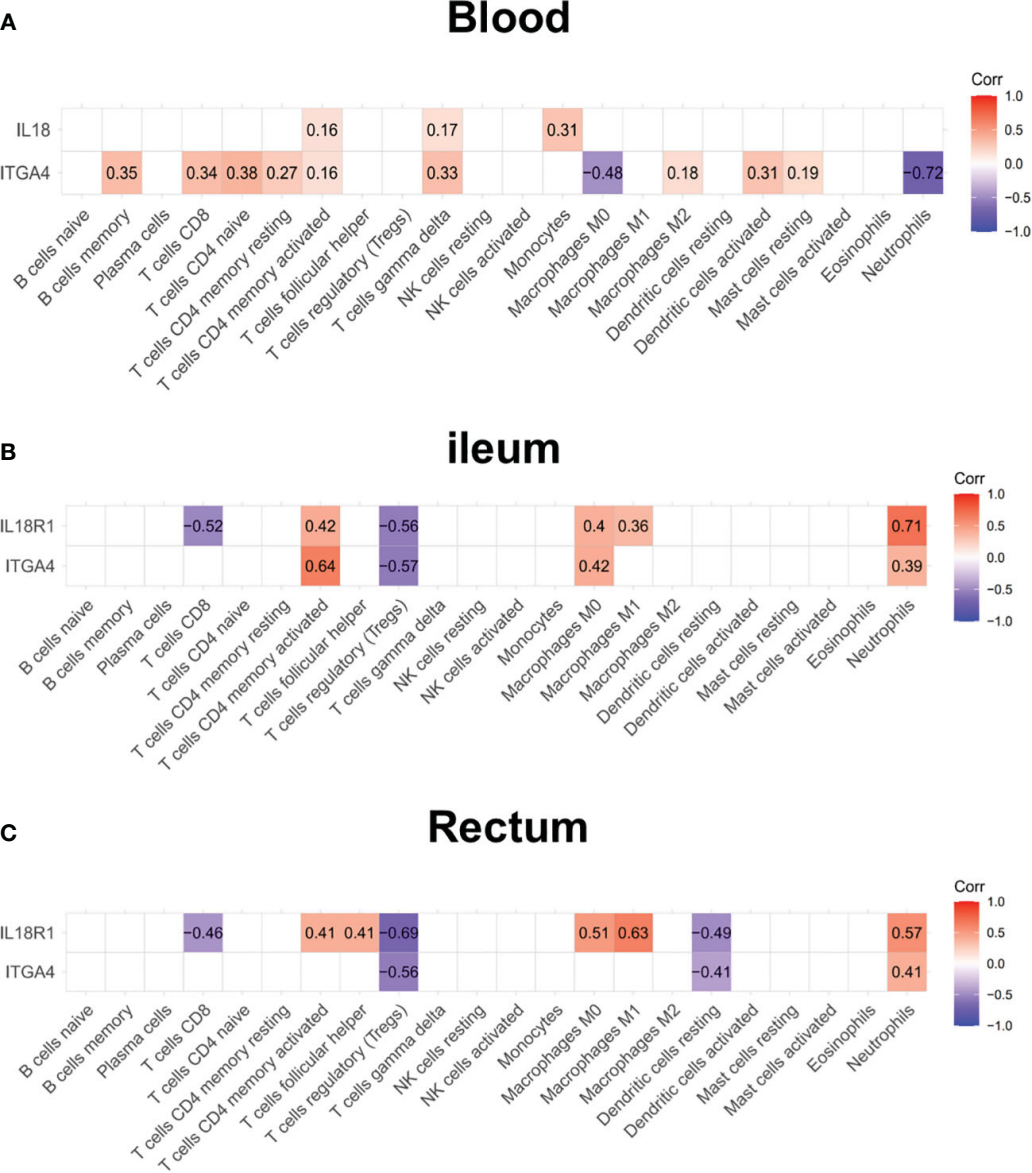
The expression of *ITGA4* and *IL18R1* in the blood and intestinal samples. The correlation analysis between the expression of *ITGA4* and *IL18R1* and immune cell abundance in the blood **(A)**, ileum **(B)**, and rectum **(C)**.

Patients with IBD have a disrupted mucosal barrier and therefore microbial products may enter the circulation, which can serve as an indicator for understanding the changes between responders and non-responders. After analyzing GSE112366 and GSE207022, which reflect gene expression in intestinal tissue, we downloaded GSE207465 from GEO to analyze blood samples and compare them with the intestinal tissue findings. GSE207465 includes 246 samples from responders and 161 samples from non-responders to ustekinumab treatment at week 8. A total of 1,125 DEGs were identified in GSE207465, namely, 251 upregulated genes and 874 downregulated genes. *ITGA4* was the only DEG that decreased in the ileum, rectum, and blood in the response group compared with the non-response group (**Figures 7A–D**). We also found that the expression of *IL18R1* in the ileum and rectum was lower in the response group than in the non-response group (**Figures 7C, D**). Furthermore, the expression of *IL18* (the ligand of *IL18R1*) was lower in the response group than in the non-response group in blood (**Figure 7B**). For further exploration, we analyzed the correlation between co-expression genes and immune cells. The results showed that co-expression genes were mainly expressed in T cells and neutrophils (see **Figures 8A–C**).

### 3.5 Differences in expression of inflammation-related genes and fibrosis-related genes before and after treatment with ustekinumab

**Figure 9:**
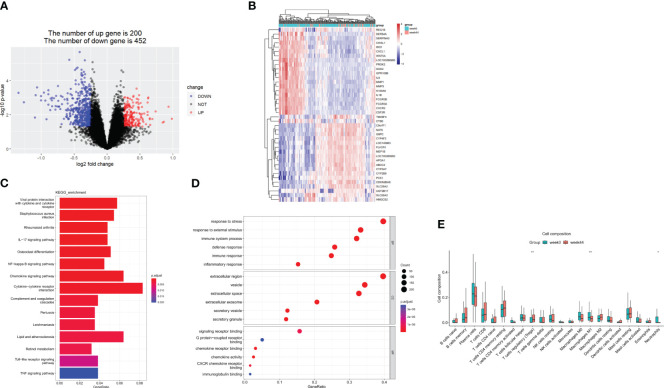
DEGs before and after ustekinumab treatment. **(A)** A volcanic map of the DEGs in the GSE112366 dataset. **(B)** A heatmap of the top 40 DEGs. **(C)** The KEGG analysis. **(D)** The GO enrichment analysis of DEGs in BP, CC, and MF. **(E)** The estimation of the infiltration of immune cells using the CIBERSORT algorithm. Wilcoxon tests were used for statistics. *p <0.05, **p < 0.01. DEGs, differentially expressed genes; GO, Gene Ontology; KEGG, Kyoto Encyclopedia of Genes and Genomes.

GSE112366 was divided into two groups including 141 samples at week 0 and 48 samples at week 44; the patients without ustekinumab treatment were excluded. A total of 652 DEGs were visualized in a volcano plot (**Figure 9A**), and the top 20 upregulated and downregulated genes were shown in a heatmap (**Figure 9B**). As shown in the heatmap, *IL1B*, *CXCL1*, *GPR109B*, *IL8*, *MMP1*, *MMP3*, and *S100A8*, which were thought to be related to inflammatory response, were changed significantly (**Figure 9B**). As shown in the KEGG analysis, the IL-17 signaling pathway, NF-kappa B signaling pathway, cytokine–cytokine receptor interaction, Toll-like receptor signaling pathway, and TNF signaling pathway were enriched in the DEGs (**Figure 9C**). BP analysis, which is one of the GO functional analyses, showed that the immune and inflammatory responses were in the top position (**Figure 9D**). Furthermore, after analyzing the proportion of immune cells between week 0 and week 44, we found that there were less neutrophils, less M1 macrophages, and more Tregs in the ileum after treatment with ustekinumab than before treatment (**Figure 9E**).

**Figure 10:**
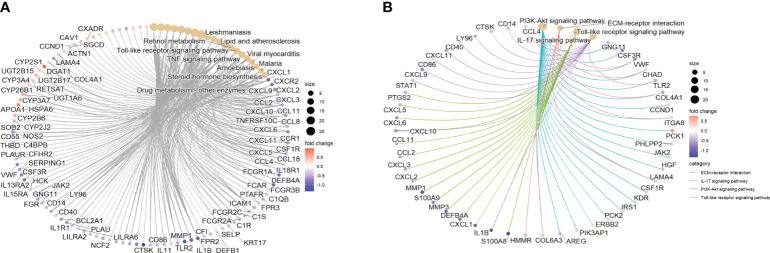
**(A)** The top 10 pathways from KEGG analysis and the genes enriched in the pathways are visualized. **(B)** The fibrosis-related pathways with significant changes and the genes enriched in the pathways are visualized. KEGG, Kyoto Encyclopedia of Genes and Genomes.

**Figure 11:**
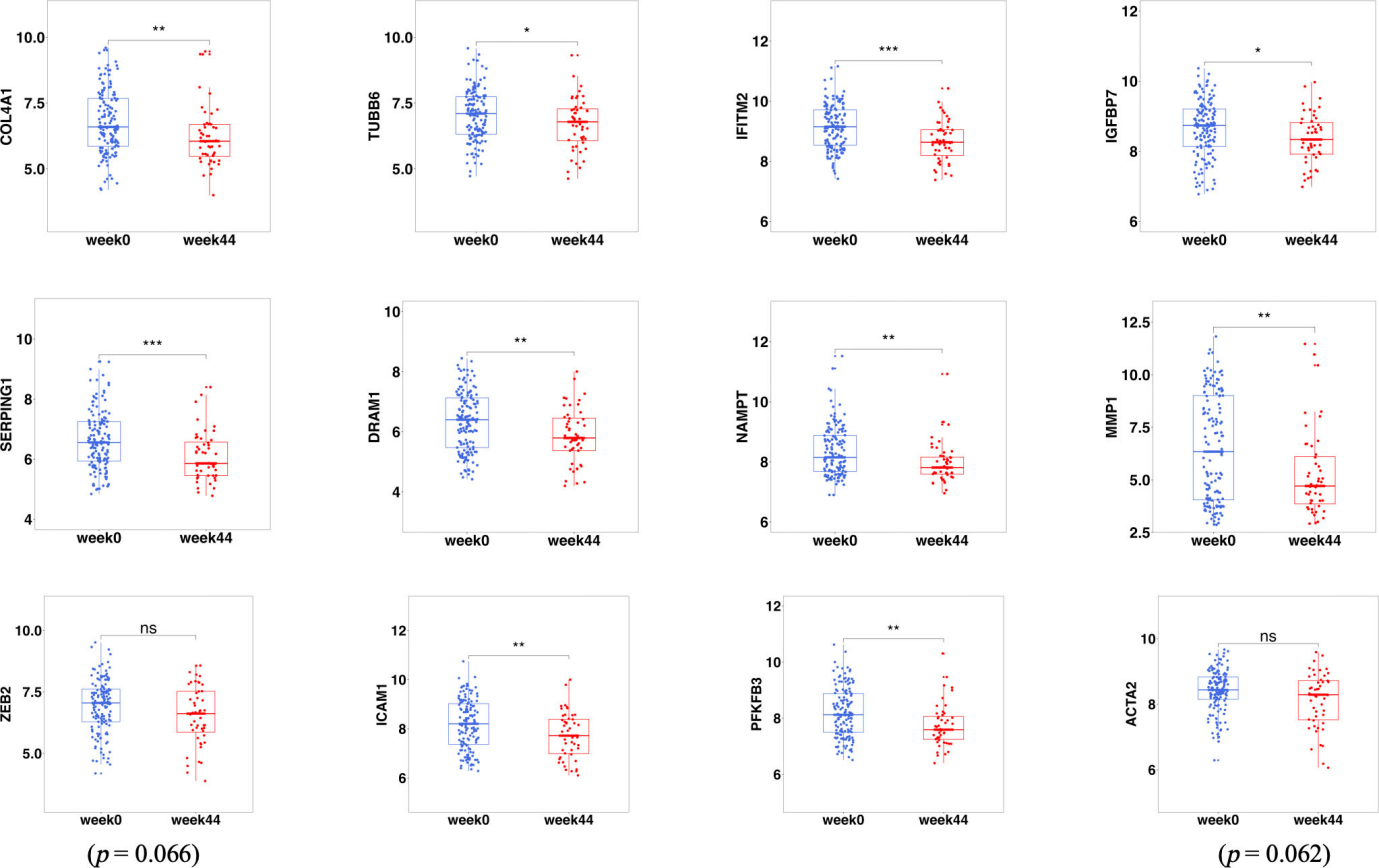
The fibrosis-related DEGs before (week 0) and after treatment (week 44) with ustekinumab. DEGs, differentially expressed genes. *p < 0.05; **p < 0.01; ***p < 0.001; ns, not significant.

To analyze the effect of ustekinumab on fibrosis in patients with CD, we explored the changes of fibrosis-related genes and pathways before and after ustekinumab treatment. The pathway analysis, shown in **Figure 10**, revealed that the fibrosis-related pathways, such as ECM–receptor interaction (*p* = 0.045) and the PI3K-AKT pathway (*p* = 0.024), were also enriched in the DEGs. In addition, *COL4A1*, which is related to ECM–receptor interaction (**Figure 10B**), was one of the markers of fibrosis and was decreased after treatment with ustekinumab (**Figure 11**). Then, we explored the fibrosis-related module genes before and after treatment with ustekinumab (26). This module was constructed in the work of Dovrolis et al., as previously referenced, and it consisted of specific CD ileal fibrosis-related genes. Afterwards, we analyzed the changes of fibrosis-related module genes before and after treatment. The expression levels of *COL4A1*, *TUBB6*, *IFITM2*, *SERPING1*, *DRAM1*, *NAMPT*, *MMP1*, *ZEB2*, *ICAM1*, *PFKFB3*, and *ACTA2* were lower after treatment with ustekinumab (**Figure 11**).

## 4 Discussion

CD is a progressive disease that can lead to the formation of complications such as abscesses and strictures. This situation seriously influences patient’s quality of life. Currently, the therapeutic medication for moderate–severe CD includes corticosteroids, immunosuppressants, and biologicals (1). The introduction of monoclonal antibodies against TNF-α (anti-TNFs) has significantly improved the treatment of CD. However, a considerable proportion of patients with CD either failed to respond or experienced a loss of response to these agents over time (27).

Ustekinumab is a new monoclonal antibody that binds with high affinity to the p40 subunit of human IL-12 and IL-23. IL-12 and IL-23 are produced by dendritic cells and macrophages (28). These two cytokines mainly affect Th1 and Th17 cells, which are important in the pathogenesis of CD. IL-12 and IL-23 can also affect NK cells, ILC1 cells, and ILC3 cells (28). Ustekinumab has been shown to be effective in the treatment of CD (8–10). However, up to 30% of patients with CD have no response to initial treatment (29). The immune response in the gut is complex and the prediction of response to treatment is helpful in the selection of therapy (30). However, there are limited studies on the predictors of treatment response to ustekinumab.

We downloaded three GEO datasets of ustekinumab treatment in CD. Firstly, we analyzed the DEGs in blood samples (GSE207465), ileal samples (GSE112366), and rectal samples (GSE207022), comparing ustekinumab responders and non-responders. Responder prediction analysis was performed on samples at week 0 (prior to drug administration). Response status was identified at week 8 post-ustekinumab treatment for ileal samples by clinical response (a decrease of 100 points from their baseline value or a value <150 by CDAI) and rectal samples by endoscopy examination of mucous membrane recovery. *MUC1*, *DUOX2*, *LCN2*, and *PDZK1IP1* were identified as hub genes in the difference between ustekinumab responders and non-responders in ileal samples, with all genes having significantly lower expression in responders. Rectal samples were then used to confirm our findings in the ileal samples. We observed that *MUC1*, *LCN2*, and *PDZK1IP1* were also lower in the response group compared with the non-response group. The RF prediction model based on these three genes and AUC–ROC analysis was then performed and indicated that *MUC1*, *LCN2*, and *PDZK1IP1* have a good performance in predicting response to ustekinumab. These results suggested that *MUC1*, *LCN2*, and *PDZK1IP1* may be involved in treatment response to ustekinumab and could serve as a predictive test to identify which patients will respond to ustekinumab treatment, saving patients’ time and money, and preventing patients from suffering.

*MUC1* is a transmembrane mucin glycoprotein that is expressed on the apical surface of mucosal epithelial cells and hematopoietic cells (31). *MUC1* expression can be modulated by inflammatory cytokines such as interferon γ (IFN-γ) and TNF-α and is related to the NF-κB pathway (32, 33). MUC1 has a protective effect on the epithelium; however, recurrent inflammation has been shown to increase the level of the hypo-glycosylated form of MUC1 (32). This form of MUC1 can increase chemotaxis of innate inflammatory cells, driving increased immune cell recruitment and inflammation. Anti-TNF-α therapy might be effective in lowering the expression of pro-inflammatory MUC1 (32). The study also shows that continued high-level expression of MUC1 may be an early biomarker for resistance to anti-TNF-α therapy (34). In addition, increased levels of MUC1 have been shown to be associated with more severe endoscopic recurrence scores (34). In our study, we found that the patients who respond to ustekinumab treatment had lower expression of *MUC1* in both the ileum and rectum. We postulate that low *MUC1* levels may indicate lower baseline inflammation and less recurrence, which may account for the susceptibility to treatment response in this subset of patients and may serve as a potential route for future investigations.

*LCN2*, also called neutrophil gelatinase–associated lipocalin (NGAL), is a potent bacteriostatic glycoprotein stored in neutrophil granules and released at sites of inflammation (35). Previous research also observed that LCN2 may be regarded as a disease activity marker of ulcerative colitis (36). The cytokines IL-17A and IL-22 are secreted by Th17 cells and induce the activation of transcription factor NF-κB, which is required for LCN2 transcription. Earlier studies have shown that LCN2 is significantly elevated in CD (37, 38). In our study, we saw that the expression of *LCN2* was lower in patients who responded to ustekinumab treatment and may predict the treatment response to ustekinumab. The possible mechanism of *LCN2* expression affecting ustekinumab treatment may be related to the effect of ustekinumab on Th17 cells (39).

*PDZK1IP1* is found to be over-expressed in patients with CD compared to healthy patients (40). Royce et al. demonstrated a strong correlation between *PDZK1IP1* expression and inflammatory cytokines, including TNF-α, IFN-γ, IL-6, and IFN-β. *PDZK1IP1* is also related to the NF-κB and STAT3 pathways (41). *PDZK1IP1* stimulates the sodium-dependent uptake of mannose and glucose through the regulation of the sodium-glucose linked transporter (SGLT) family (41). Moreover, *PDZK1IP1* is known to induce the differentiation of monocytes to dendritic cells and regulates the immune microenvironment (42). Thus, lower expression of *PDZK1IP1* in ustekinumab treatment responders than non-responders in our study may be associated with different immune microenvironments.

Current studies have shown that the neutrophil–lymphocyte ratio (NLR), which can reflect the status of inflammation in a disease, can predict the loss of response of infliximab therapy (43). The results in our study also showed that the ileum of responders contained more Tregs than non-responders. Patients had less neutrophils, less M1 macrophages, and more Tregs in the ileum after ustekinumab treatment. In addition, *MUC1*, *LCN2*, and *PDZK1IP1* were mainly expressed in neutrophils and lymphocytes. In the GO analysis, these hub genes we identified were related to DNA damage, ferroptosis, and oxidative stress, which is related to inflammation, potentially indicating how the hub genes mediate the therapeutic response. Nowadays, the target of CD treatment has shifted from clinical response to mucous healing (11). *MUC1*, *LCN2*, and *PDZK1IP1* had a good performance in predicting ustekinumab responders not only for clinical response (GSE112366) but also in mucous healing (GSE 207022).

We wanted to figure out the relationship between the expression changes in the ileum, rectum, and blood. We found that *ITGA4* is changed both in the blood and in intestinal tissues. *IL18* is lower in blood while *IL18R1* is lower in intestinal tissues. *ITGA4* is a well-known α4 integrin that is expressed on lymphocytes and is related to lymphocyte trafficking into the intestine (44). In our study, we analyzed the relative gene expression of *ITGA4* and *IL18R1* in immune cell populations. *ITGA4* is related to T cells in the gut and neutrophils in the blood. A study showed that *ITGA4* could also be expressed on the surface of neutrophils (45). Neutrophils and lymphocytes play an important role in the treatment response of ustekinumab (46). IL‐18, a member of the IL‐1 family, is similar to IL‐1β (47). In homeostatic conditions, IL‐18 is protective, but in pathological states, it is involved not only in the activation of Th1 and NK cells, but also in the activation of Th2, IL‐17‐producing γδT cells, and macrophages (47). In addition, it is associated with the TLR-MyD88 and NF-κB pathways. Further studies should be done to investigate the expression of IL-18 in patients with ustekinumab.

Intestinal fibrosis is an important complication of CD (4), the pathogenesis of the fibrosis is still unclear, and it may be related to some immune cells, such as Th17 cells (4). It is well known that ustekinumab can affect Th17 and Th1 cells, but there are few studies on whether ustekinumab has an anti-fibrotic effect. We wanted to explore the effect of ustekinumab on inflammation and fibrosis. In an inflammation-related analysis, we found that the IL-17 signaling pathway, NF-kappa B signaling pathway, cytokine–cytokine receptor interaction, Toll-like receptor signaling pathway, and TNF signaling pathway were changed after treatment with ustekinumab, as well as some inflammatory genes like *IL1B*, *CXCL1*, *GPR109B*, *IL8*, *MMP1*, *MMP3*, and *S100A8.* Some hub genes are associated with the NF-κB pathway, as we mentioned previously; thus, the NF-κB pathway may play an important role in the treatment response of ustekinumab. In a fibrosis-related analysis, we found that pathways like the ECM–receptor interaction (*p* = 0.045) and the PI3K-AKT pathway (*p* = 0.024), which were associated with fibrosis, were changed after treatment. The fibrosis module genes were investigated and we found that an extracellular matrix component, COL4A1, was decreased after treatment, and other fibrosis-related genes (*TUBB6*, *IFITM2*, *SERPING1*, *DRAM1*, *NAMPT*, *MMP1*, *ZEB2*, *ICAM1*, *PFKFB3*, and *ACTA2*) were also decreased after ustekinumab treatment. Ustekinumab may have an effect on alleviating fibrosis.

The limitation of our study is that the expression of hub genes was not validated in new patients but will be verified in the future. Moreover, our analysis of the effect of ustekinumab on fibrosis is only at the genetic level and will need to be further validated by cohort studies in the future. However, we hope that our study can provide a basis for further research.

In conclusion, *MUC1*, *LCN2*, and *PDZK1IP1* are the hub genes in gut associated with ustekinumab response. The changes in the expression of *ITGA4* and *IL18/IL18R1* both in blood and in the gut might play an important role in the response to ustekinumab. Ustekinumab may have an impact on fibrosis.

## Data availability statement

The original contributions presented in the study are included in the article/**Supplementary Material**. Further inquiries can be directed to the corresponding author.

## Author contributions

YX: Formal Analysis, Writing – original draft. SW: Visualization, Writing – original draft. ZY: Software, Validation, Writing – original draft. HZ: Writing – review & editing.
