# Supplementary figures and images for: Identifying hub genes in response to ustekinumab and the impact of ustekinumab treatment on fibrosis in Crohn’s disease
Source: Front Immunol. 2024 May 22;15:1401733. doi: 10.3389/fimmu.2024.1401733 (PMC11150586; doi:10.3389/fimmu.2024.1401733)

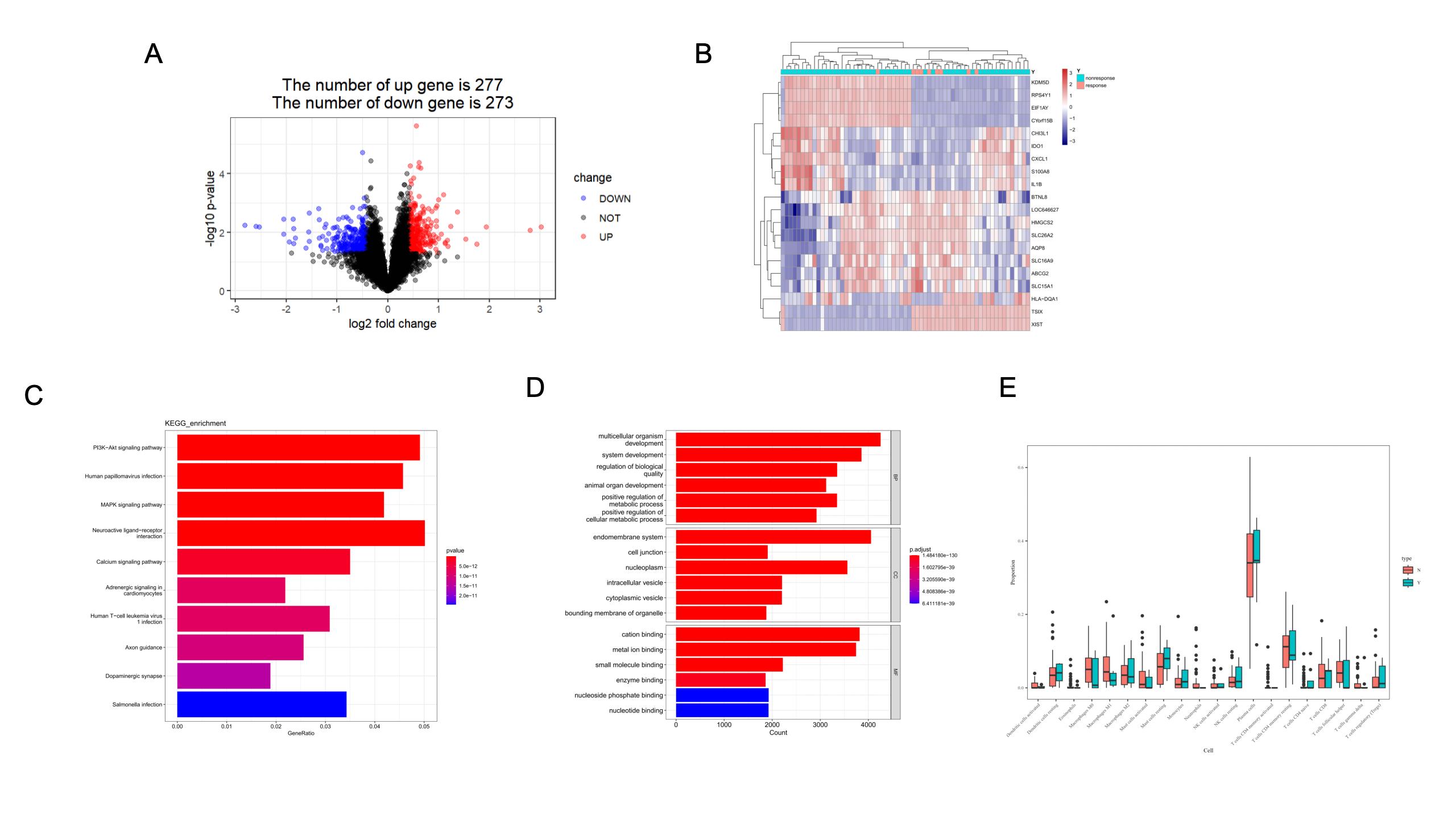

Supplement: Supplementary file 1 [file DataSheet_1.zip › raw data/supplymentary figure/GSE207022.jpg]
